# Divergence of transcriptional landscape occurs early in B cell activation

**DOI:** 10.1186/s13072-015-0012-x

**Published:** 2015-05-14

**Authors:** Trent Fowler, Alexander S Garruss, Amalendu Ghosh, Supriyo De, Kevin G Becker, William H Wood, Matthew T Weirauch, Stephen T Smale, Bruce Aronow, Ranjan Sen, Ananda L Roy

**Affiliations:** Department of Developmental, Chemical and Molecular Biology, Sackler School of Biomedical Science, Tufts University School of Medicine, 150 Harrison Avenue, Boston, MA 02111 USA; Wyss Institute for Biologically Inspired Engineering, Harvard University and Department of Genetics, Harvard Medical School, Boston, MA 02115 USA; Laboratory of Molecular Biology and Immunology, National Institute on Aging, Baltimore, MD 21224 USA; Gene Expression Unit, Laboratory of Genetics, National Institute on Aging, Baltimore, MD 21224 USA; Center for Autoimmune Genomics and Etiology (CAGE) and Divisions of Biomedical Informatics and Developmental Biology, Cincinnati Children’s Hospital Medical Center, Cincinnati, OH 45229 USA; Department of Microbiology, Immunology, and Molecular Genetics, UCLA, Los Angeles, CA 90095 USA

## Abstract

**Background:**

Signaling via B cell receptor (BCR) and Toll-like receptors (TLRs) results in activation of B cells with distinct physiological outcomes, but transcriptional regulatory mechanisms that drive activation and distinguish these pathways remain unknown.

**Results:**

Two hours after ligand exposure RNA-seq, ChIP-seq and computational methods reveal that BCR- or TLR-mediated activation of primary resting B cells proceeds via a large set of shared and a smaller subset of distinct signal-selective transcriptional responses. BCR stimulation resulted in increased global recruitment of RNA Pol II to promoters that appear to transit slowly to downstream regions. Conversely, lipopolysaccharide (LPS) stimulation involved an enhanced RNA Pol II transition from initiating to elongating mode accompanied by greater H3K4me3 activation markings compared to BCR stimulation. These rapidly diverging transcriptomic landscapes also show distinct repressing (H3K27me3) histone signatures, mutually exclusive transcription factor binding in promoters, and unique miRNA profiles.

**Conclusions:**

Upon examination of genome-wide transcription and regulatory elements, we conclude that the B cell commitment to different activation states occurs much earlier than previously thought and involves a multi-faceted receptor-specific transcriptional landscape.

**Electronic supplementary material:**

The online version of this article (doi:10.1186/s13072-015-0012-x) contains supplementary material, which is available to authorized users.

## Background

B cell activation, the transition from a naïve to an effector state, is important due to its essential role in immunity. Deregulated activation can have disastrous effects resulting in immune disorders and several B cell malignancies, some of which resemble activated B cell phenotypes [1, 2]. Mature resting splenic B cells maintain a quiescent G_0_ state with limited proliferative output [3]. Upon encountering antigen, these cells become activated, leading to plasma cell differentiation and participation in immune responses. Activation of B cells can occur through either surface B cell receptor (BCR) [4–6] or various pathogen-associated molecular patterns such as bacterial lipopolysaccharide (LPS), which is mediated by Toll-like receptor (TLR) signaling and NF-κB pathways [7]. Exposure of B cells to LPS via TLR4 can promote plasma cell differentiation [7, 8]. A properly regulated LPS activation appears critical as patients with deficient TLR signaling molecules, exhibit autoimmunity [9].

Splenic B cell differentiation can begin as early as 4 h and fully develop by 48–72 h [10]. While much is known about signaling cascades during B cell activation at early and late time points [5, 11, 12], transcriptional changes during these times are still being addressed [13]. In particular, a high-resolution picture reflecting the immediate transcriptional and epigenetic changes during early B cell activation, before mature B cells proceed toward proliferation and functional immune responses occur, is not available. Regardless of cell type, initial signaling events lead to rapid induction of primary response genes (PRGs) whose products initiate secondary waves of transcription resulting in egress from the G_0_ state and subsequently in proliferation and effector function [14]. Regulatory mechanisms for these rapid responses, release of preformed “paused” transcription complexes, RNA polymerase II (Pol II) recruitment via transcription co-factors, and promoter accessibility/repression through histone modifications, are particularly well established [14, 15]. But how these mechanisms operate during BCR and LPS signaling in early activation of B cells is not known.

Activation of resting splenic B cells ex vivo provides a tractable model to explore this transition in a ligand-specific manner [16]. B cells are unique hematopoietic cells because they express both BCR and TLRs. Although stimulation of naïve ex vivo B cells through both receptors elicits activation and proliferation, only LPS stimulation results in plasma cell differentiation [4, 17, 18]. We used this ex vivo model to determine how and when these ligand-specific transcriptional landscapes may diverge. We observe clear differences within 2 h post stimulation. In addition to ligand-selective differences in both protein-coding and non-coding RNAs, several other transcriptional regulatory steps differed between the activation states providing three key findings [1]. Although BCR-induced genes show new recruitment of RNA Pol II that appeared to be paused at promoters, LPS/TLR4-induced genes exhibit enhanced transition of RNA Pol II from initiation to elongation [2]. While the H3K4me3 (activating) mark is increased in both activation states (more so during TLR4 engagement), the appearance of the H3K27me3 (repressive) mark is reduced on BCR-responsive genes but remains relatively unchanged in LPS-responsive genes [3]. Predicted transcription factor binding sites in the promoter proximal regions of genes also differ significantly in a ligand-selective manner. Together, our results show that B cell egress from the resting state involves a large pool of shared/common RNAs, and a small set of signal-selective RNAs that exhibit remarkable transcriptional landscape changes soon after ligand engagement.

## Results

### Response dependent differential transcription during early activation

**Fig. 1:**
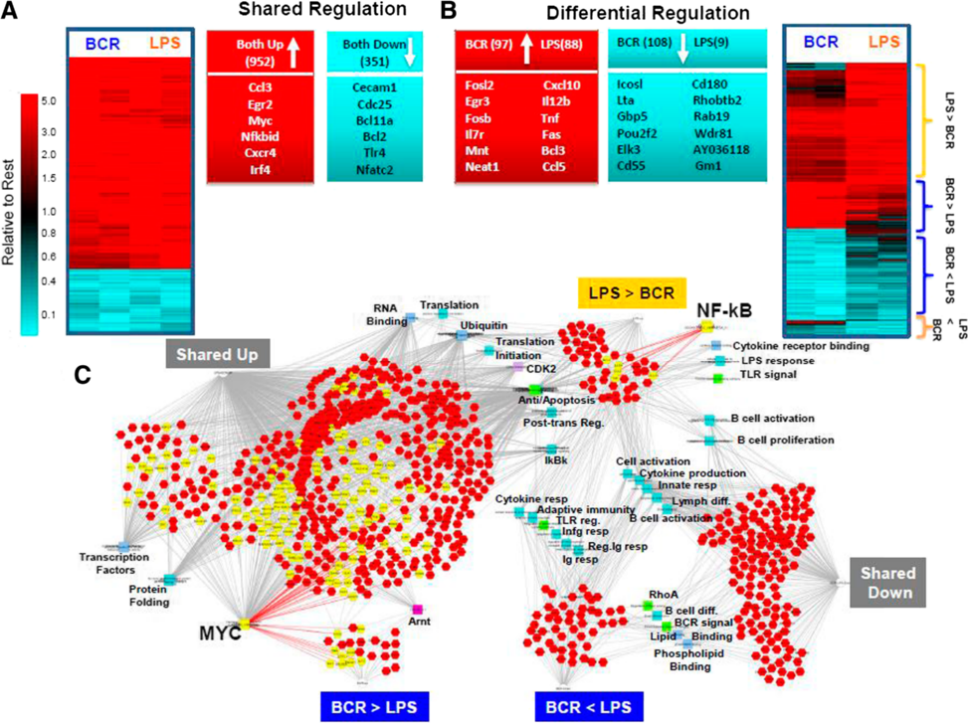
Differential transcription regulation signatures by two B cell activation modes. Clustered heatmaps of RNA-seq data show genes with increased (*red*) or decreased (*blue*) transcription shared by both stimulated cell types (**a**) or preferred during either response; for example, BCR > LPS, see Methods for further explanation (**b**). **c** Gene lists associated within each cluster are represented by heatmaps shown in (**a**) and (**b**) and their relationship to processes were analyzed for significant, *P* < 0.05, relationships. For each gene (*red*) in a differential expression cluster, e.g. BCR > LPS, an association with a particular processes or regulatory element is indicated with a *line*. Myc and NF-κB, along with the genes, processes and regulatory elements directly associated with them are shown in *yellow*

To determine how naïve B cells proceed along activation pathways in response to different signals, high-resolution RNA-seq analysis was performed after 30 and 120 min of stimulation with anti-IgM (engages BCR) or LPS (engages TLR4). Many expected genes, including the early activation marker *CD69* and the PRGs c-*fos*, c-*jun*, and c-*myc*, were increased compared to the resting state by exposure to either anti-IgM/BCR or LPS/TLR4 as early as 30 min (Additional file 1: Figure S1). However, 120 min was required to identify genes/transcripts of the BCR and TLR4/NF-κB signaling pathways with robust *P* values (Additional file 1: Figure S1). Therefore, all subsequent experiments were performed at 120 min. The majority of the differentially expressed genes were shared between BCR and LPS activation henceforth referred to as shared response genes (Fig. 1a), which expectedly include c-*myc*, *egr2*, and *irf4*, along with more immune specific genes, *ccl3*, and *cxcr4* (Fig. 1a). Smaller subsets of response selective transcripts were induced in each of the activated states with corresponding relevance to their specific stimuli (Fig. 1b). For example, BCR preferentially increased *il-7r*, which is associated with B cell development and differentiation [19], while LPS increased the inflammatory cytokines *cxcl10* and *ccl5* [20]. Surprisingly, very few [9] genes were decreased in the LPS response. The significance of this observation remains unclear but this paucity of data precluded any meaningful comparison of a ligand-specific decrease in transcription. Therefore, subsequent analyses focused solely on induced mRNAs.

Figure 1c shows functional relationships between the different groups of genes with shared and response selective transcription that changed significantly relative to the resting state. c-Myc, which plays an important role in B cell proliferation in response to TLR4 and BCR ligation, was dominantly positioned in the shared response genes and also prevalent with BCR-selective genes (known Myc targets are highlighted in yellow at bottom left). As expected, preferentially increased LPS specific transcripts contained TLR signaling pathway genes and known NF-κB targets (Additional file 2). We conclude that the initial stages of B cell activation involve a large set of shared genes, despite being stimulated by distinct signaling pathways and a small but significant set of ligand-selective genes. These ligand-selective genes induced by BCR showed a predominant Myc signature, while those induced by TLR4 exhibited a prominent NF-κB signature.

We also analyzed 1315 genes whose transcription was not significantly altered by BCR or TLR4 stimulation. These genes, such as Polr2a, Max, ActB, and Dicer1, collectively annotated to biological processes, including maintenance of homeostasis, cell cycle, and apoptosis management (Additional file 1: Figure S2). In addition, transcripts associated with antigen processing, TGF-β signaling, TNF signaling, and MHC1 antigen presentation, were also unchanged (Additional file 1: Figure S2), suggesting that prior to activation, these cells are actively executing significant immune functions.

### Response dependent RNA Pol II occupancy during early activation

**Fig. 2:**
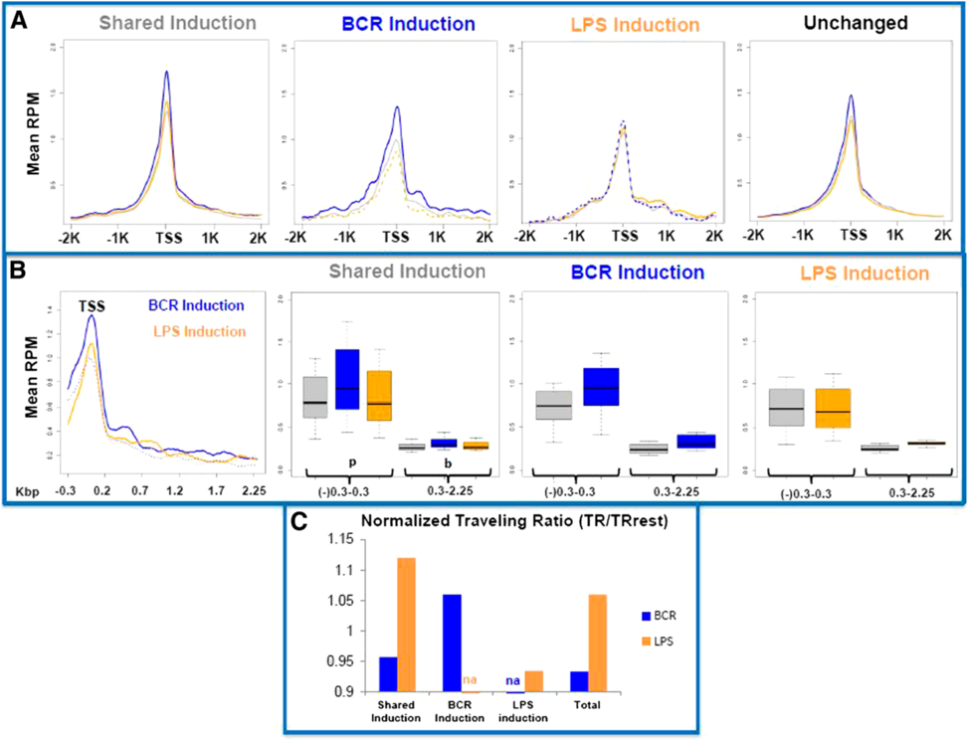
Stimulus Responsive RNA Pol II occupancy. **a** RNA Pol II occupancy normalized mean reads per million (RPM) were mapped 2 kbp relative to average TSS of genes with increased transcription shared between the two activation states (*far left*), preferential to BCR (*middle left*) or LPS (*middle right*), and unchanged transcription relative to the resting state (*far right*). Reads from the resting state are in grey, BCR in blue, and LPS in orange. **b** Analysis of promoter proximal and “gene body” RNA Pol II occupancy. Direct overlays of histographs from BCR induction and LPS induction groups, *far left*, show regions of analysis from the promoter area to further in the gene body where RNA Pol II starts from BCR (*solid blue*) and LPS (*solid orange*) starts to merge at 2250 past the TSS. Resting Pol II occupancy is shown in *dotted lines* for BCR induced genes (*blue*) and LPS-induced genes (*orange*). Boxplots contain the sums of mapped normalized mean RPM for each induced transcription group relative to average TSS; resting (*grey*), BCR (*blue*), LPS (*orange*), number of genes indicated in parentheses. Promoter proximal occupancy (p) was defined as 0.3 kbp before and after the TSS while gene body occupancy was defined from 0.3 to 2.25 kbp past the TSS. **c** Traveling ratios of Pol II occupancy were determined to describe average RNA Pol II movement between the promoter and interior of the gene. Ratios of mean RPMs shown at *black line* in boxplot from (**b**) were calculated; traveling ratio (TR) = (RPM mean) body(b)/(RPM mean) promoter(p). Here, we report TR relative to the resting state (TRactivation/TRrest). TRs for each cellular state; rest, BCR, and LPS are shown in Supplemental Fig. S4B

To examine the transcriptional status of these genes, we investigated RNA Pol II occupancy at the promoters of both shared and preferentially induced genes. While RNA Pol II occupancy increased in genes, exhibiting both induced and unchanged transcription, a significant genome-wide increase in RNA Pol II recruitment to TSS(s) during BCR activation was also observed (Fig. 2a and Additional file 1: Figure S5). Statistical analysis (Additional file 1: Figure S4) indicated this difference was significant and not due to high variability. RNA Pol II occupancy did not increase during LPS activation at induced and unchanged genes, though the small increase in downstream RNA Pol II at genes preferentially induced by LPS suggested promoter-associated (paused) RNA Pol II was transiting to regions downstream of TSS upon LPS stimulation (Fig. 2a). These events occurred whether considering occupancy around the TSS(s) of all possible transcripts (Fig. 2) or at the TSS with the highest RNA Pol II occupancy (Additional file 1: Figure S3). To better determine the difference in downstream RNA Pol II occupancy between the two signals and whether these differences reflect transition from initiation to elongation, we calculated RNA Pol II traveling ratios (TR) [21, 22]. Both downstream analysis (Fig. 2b) and TR (Fig. 2c) clearly show that RNA Pol II occupancy downstream of TSS is significantly different between the two signaling pathways. However, because this assay measures the whole RNA Pol II population, it is not possible to conclusively determine whether RNA Pol II containing transcriptional complexes transition at the same or different rates. Nevertheless, these data collectively suggest that while genes induced by BCR recruited additional RNA Pol II, the transition from initiation to elongation is less, suggesting a pausing mechanism. In contrast, although the RNA Pol II association at the promoter was lower with LPS-induced genes, LPS activation results in a more robust transition from initiation to elongation mode.

### Response dependent changes in chromatin

**Fig. 3:**
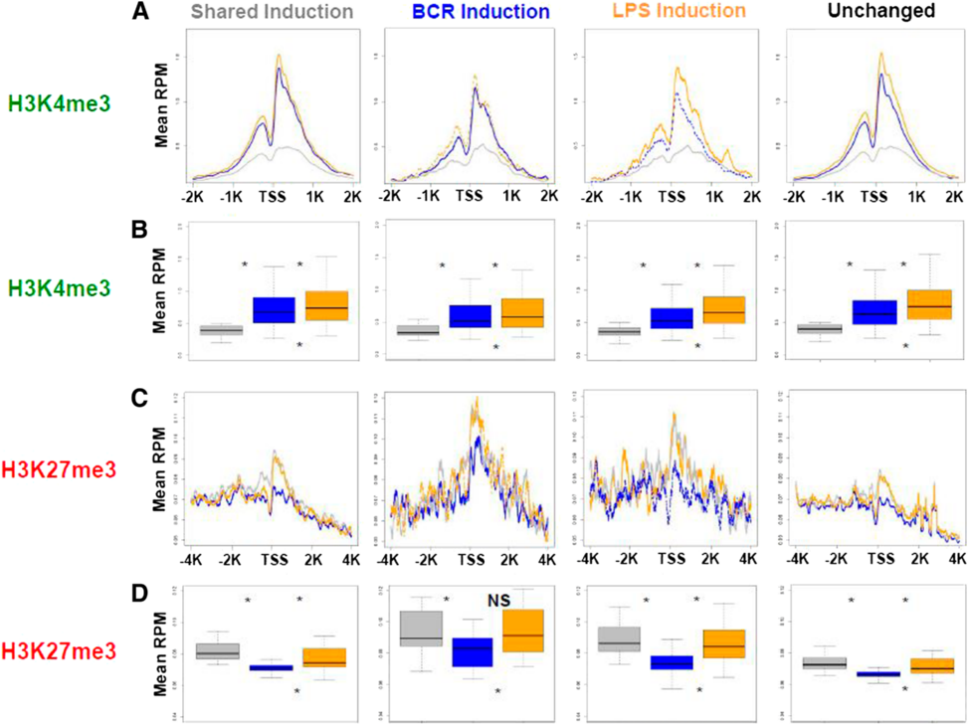
Differential response of histone marks. **a** H3K4me3 normalized mean RPM were mapped 2 kbp relative to average TSS of genes with increased transcription shared between the two activation states (*far left*), exclusive to BCR (*middle left*), exclusive to LPS (*middle right*), and in genes with substantial but unchanged transcription relative to the resting state (*far right*). Reads from the resting state are in grey, BCR in blue, and LPS in orange. **b** Boxplots contain the sums of mapped H3K4me3 normalized mean RPM +/−1 kbp relative to average TSS; resting (*grey*), BCR (*blue*), LPS (*orange*), and number of genes indicated in parentheses. Non-significant (NS) (*P* > = 0.05) or significant (*, *P* < 0.05) from Wilcoxon Rank Sum Testing. *Asterisk* or *NS* on top indicates a difference from the resting state and at the bottom between the BCR and LPS states. **c** H3K27me3-normaliized mean reads per million 4 kb on either side of average TSS of genes. **d** Boxplots contain the sums of mapped H3K27me3 normalized mean RPM +/−1 kb relative to average TSS; resting (*grey*), BCR (*blue*), LPS (*orange*), and number of genes indicated in parentheses. Non-significant (NS) (*P* > = 0.05) or significant (*, *P* < 0.05) from Wilcoxon Rank Sum Testing. *Asterisk* or *NS* on top indicates a difference from the resting state and at bottom between the BCR and LPS states

Chromatin modification is a well-defined transcriptional regulatory mechanism [14, 23]. Increased tri-methylation of promoter proximal histone 3 lysine 4 (H3K4me3) is associated with enhanced transcription complex recruitment and retention [24]. A measurable level of H3K4me3 was observed in the resting state, which was further increased upon signaling through either BCR or TLR4. However, compared to BCR activation, LPS signaling via TLR4 increased H3K4me3 at genes with unchanged transcription (Fig. 3a, b) and in a genome-wide manner (Additional file 1: Figure S5). Increase in H3K4me3 was further enhanced at genes preferentially induced by LPS. While many of the genes regulating deposition of H3K4me3 are known [24], none were differentially transcribed in either BCR or TLR4 activation. We conclude that while increased H3K4me3 is associated with B cell activation in general, it is more pronounced at LPS/TLR4 responsive genes than at BCR-responsive genes.

Polycomb repressor complexes (PRCs) catalyze repressive chromatin marks [25–28]. In mammals, PRC2 activity depends on the SET domain-containing protein Enhancer of zeste homolog 2 (EZH2) that catalyzes transcriptionally repressive histone H3 methylation at lysine 27 mark (H3K27me3) [29]. H3K27me3 plays an important role in B cell proliferation, and EZH2 expression is low in resting B cells but upregulated in activated B cells [30–32]. Hence, we analyzed H3K27me3 in response to BCR and TLR4 engagement. In genes with increased or unchanged transcription, the level of H3K27me3 at promoters was decreased during BCR activation (Fig. 3c). This decrease was most prominent around the TSS, and while there was a small decrease during LPS activation, this was restricted to an area just upstream of the TSS; otherwise, H3K27me3 in the LPS activation state remained largely unperturbed. BCR activation showed a greater genome-wide decrease in H3K27me3 than LPS (Additional file 1: Figure S5). While the boxplot comparison of median analysis did show some variation, the differences remained statistically significant. We concluded that there was a decrease in H3K27me3 during BCR engagement relative to TLR4 engagement around TSSs. Collectively, analysis of H3K4me3 and H3K27me3 illustrates that distinct chromatin modifications separate BCR and LPS activation states, regardless of whether considering all possible transcripts (Fig. 3) or the ones with the RNA Pol II highest occupancy (Additional file 1: Figure S6) was considered.

### Analysis of individual genes

**Fig. 4:**
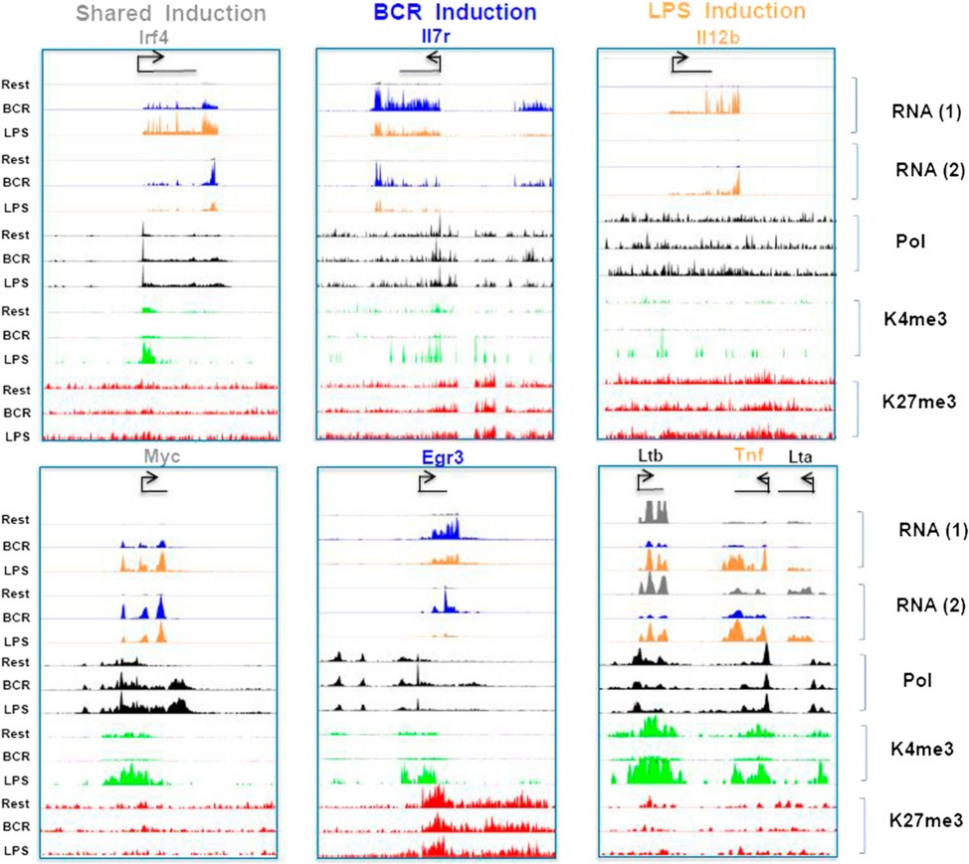
Analysis of individual genes. Normalized genome-mapped RPM visualized on custom UCSC Genome Browser tracks are shown for two genes representing the common increased (*left*), BCR exclusive increased (*middle*), and LPS exclusive increased response (*right*) are shown with levels scaled to the maximum of the given set. RNA transcription is shown for the resting (*grey*), BCR-activated (*blue*), and LPS-activated (*orange*) states along with RNA Pol II (*black*), H3K4me3 (*green*), and H3K27me3 (*red*) in the same order; resting, BCR, and LPS

Having observed ligand-selective regulatory signatures at shared and response selective genes, we examined two representative genes from each category (shared, BCR- and LPS-selective genes) based on known functions in B cell activation and immunity and on the presence or absence of a Myc signature (for the shared and BCR-selective groups) or a NF-κB signature (for the TLR4/LPS-selective group). These genes collectively illustrated the characteristics observed at the global level. For example, the shared genes (*Irf4* and *Myc*) as well as BCR-responsive genes (*Il7r* and *Egr3*) showed an increase in RNA Pol II and a decrease in H3K27me3 (Fig. 4). While *Irf4* and *Myc* showed increased RNA expression in response to both stimuli, *Il7r* and *Egr3* showed preferential RNA expression in response to BCR stimulation. Conversely, the LPS-responsive genes (*Il12b* and *Tnf*) showed a preferential increase in RNA expression and an increase in RNA Pol II at downstream sites, while the H3K27me3 mark around the TSS remained largely unchanged. Lastly, although H3K4me3 was increased by both stimuli, it was clearly more pronounced at *Il12b* and *Tnf* after LPS stimulation. Hence, the global transcriptional patterns observed with each category of genes are also observed at individual target genes.

### Response selective transcription factor binding motif enrichment

We next examined promoter sequences to gain further insights into the observed differences in RNA Pol II recruitment or regulatory histone marks. Promoters containing “CpG islands” correlate with low nucleosome occupancy and increased RNA Pol II occupancy [33–35]. A majority of transcripts was associated with promoters containing or near (within 200 bps) a predicted CpG island; however, no preference for CpG associated promoters was evident for either response (Additional file 1: Figure S8). Although a connection between H3K27me3 and CpG islands exists [36], we observed no significant ligand-dependent correlation between CpG island promoters and H3K27me3 (data not shown).

**Fig. 5:**
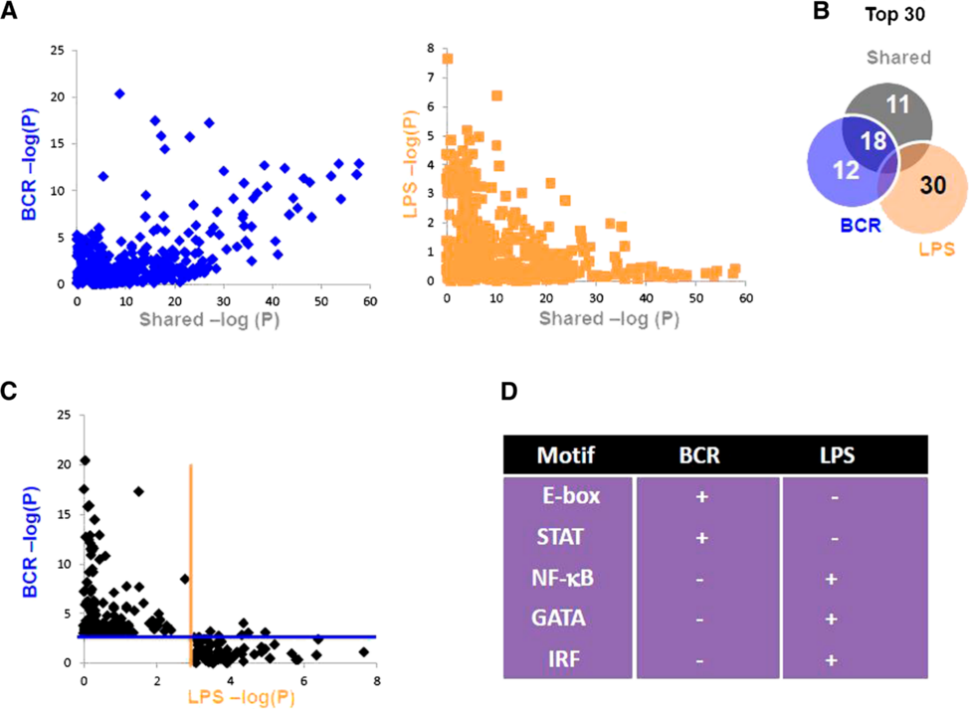
TF motif enrichment at the promoter regions. TF motif enrichment in promoters of genes associated with increased transcription was calculated using the HOMER tool (see Methods). **a** Preferential vs Shared motif *P* value comparison. Inverse log *P* values for transcription factor motifs are reported for the shared increase group (X-axis) and the BCR (*blue*) or LPS (*orange*) groups (Y-axis). **b** Motifs with the lowest 30 *P* values were taken from each group and compared via Venn Diagrams. While Shared and BCR contained 18 of the same predicted motifs, LPS overlapped neither BCR nor Shared. **c** Direct comparison of TF motif content in preferentially increased gene promoters. Inverse log *P* values with *P* < 0.001(−log(*P*) = 3) for transcription factor motifs were graphed with values for BCR on the Y-axis and LPS values on the X-axis. *Blue line* refers to BCR > 3.0 and orange line LPS > 3.0. Note that nearly all motifs are specific to only one of the two groups. **d** Summary of properties of TFs predicted to bind motifs based on the 30 motifs with the lowest *P* values

To further shed light on the signal-specific activation states, transcription factor (TF) motifs within the promoter region (−1000 to +1000 of the TSS) were examined. TF motifs at induced gene promoters were clearly different between the two activation states. Most TF motifs were highly overrepresented in both the Shared- and BCR-induced gene sets, as shown by the large number of data points along the diagonal of the scatterplot (Fig. 5a). A much smaller degree of commonality was observed between the Shared- and LPS-responsive genes where many of the diagonal data points seen in the BCR vs Shared comparison migrated closer to the axes. Taking general motifs from each data set with the 30 lowest *P* value scores (highest association), we observed that many high-frequency motifs were common between Shared and BCR, while neither overlapped with the 30 lowest *P* value motifs found in LPS preferentially induced gene promoters (Fig. 5b). Direct comparison of all significantly represented TF motifs associated with the BCR- and LPS-responsive genes showed very little overlap (Fig. 5c). The general binding properties of the TFs associated with these motifs were strikingly different, while BCR activation is associated with a preponderance of helix-loop-helix containing TFs and STAT binding sites; NF-κB, GATA, and IRF binding sites dominated the LPS-selective signature (Fig. 5d). The direct role of these TFs in recruiting RNA Pol II or epigenetic marks is unknown but that the two responses exhibit such a mutually exclusive set of TFs motifs, which is noteworthy.

### Expression of non-coding RNAs

**Fig. 6:**
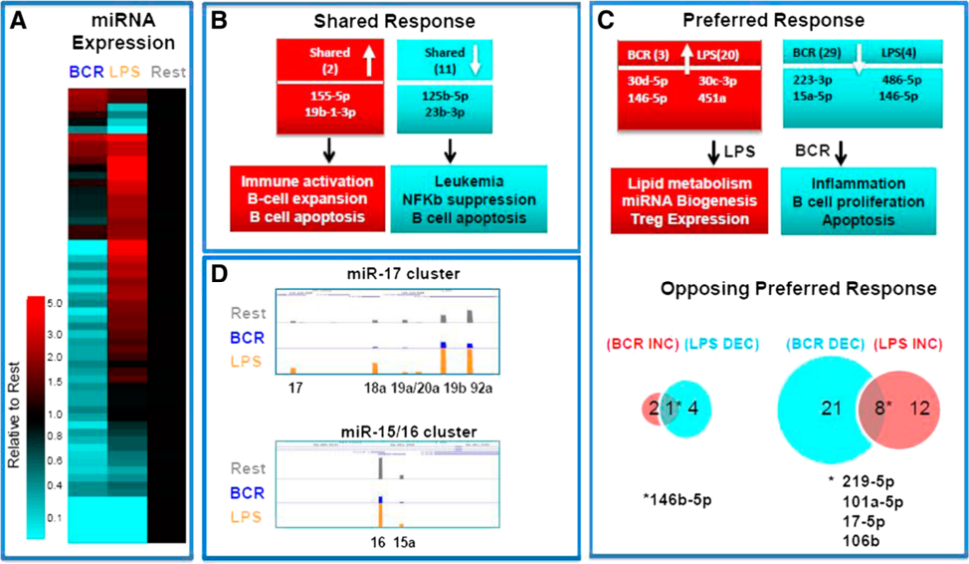
Response selective expression of miRNAs. **a** Differential expression of 60 miRNAs is shown with heatmap values indicating no change (*black*), increase (*red*), or decrease (*blue*) compared to the resting state. An increase or decrease was minimally a twofold difference. **b** miRNAs increased (*red*) or decreased (*blue*) in both BCR and LPS activation with numbers of miRNAs in parentheses, two examples from each group, and published biological effects from miRNAs in each group. **c** miRNAs preferentially increased (*red*) or decreased (*blue*) in either BCR or LPS activation with numbers of miRNAs in parentheses, two examples from each group, and published biological effects from miRNAs in each group. Venn diagrams show miRNAs common to opposing reactions to activation states; increased in BCR/decreased in LPS or decreased in BCR/increased in LPS. (*Asterisk*) miRNAs common to opposing responses with four representatives for those decreased in BCR and increased in LPS shown. **d** UCSC Genome Browser track visualization of two miRNA clusters; resting (*grey*), BCR (*blue*), and LPS (*yellow*)

Because miRNAs are known to play a pivotal role in various B cell processes such as development, inflammation, and tolerance [37, 38], we tested whether signal-selective miRNA expression could further account for the distinct transcriptional signatures. Analysis of a separate RNA sequencing set designed for improved miRNA detection revealed miRNA expression relative to the resting state that differs greatly between the two modes of activation (Fig. 6a, b). Consistent with previous observations, we found that both BCR and LPS enhanced miR-155 and miR-19b-1 expression [39, 40]. Expression of several miRNAs was decreased during BCR and LPS stimulation, including miR-125b, which when overexpressed, is capable of driving lymphoma development (Fig. 6b) [41]. Of the 60 miRNAs with significantly changed expression relative to the resting state, the majority show a difference in expression; 20 increased by LPS and 29 decreased by BCR; the full list of miRNAs can be found in Additional file 1: Figure S9 and Additional file 3: Table S1. Two expression group representatives annotated to biological processes relevant to B cell activation, including several miRNAs decreased during BCR activation and increased by LPS, are shown (Fig. 6c). Overall, we found that a far greater decrease in miRNA expression occurred during the BCR response while LPS response showed a greater induction of miRNA compared to the resting state. An interesting time-dependent transcription of two RNA editing associated long non-coding RNAs (lncRNAs), Neat1, and Malat1 [42, 43], also suggested that lncRNA management of RNA editing might provide another level of regulation during early B cell activation (Additional file 1: Figure S10).

## Discussion

Deregulation of B cell activation can result in autoimmune disorders, chronic inflammation, and lymphoma. Although B cells express both BCR and TLR4, the functional consequences to these stimuli are distinct, at least under ex vivo conditions. While BCR engagement (triggered by anti-IgM stimulation) leads to proliferative expansion of activated B cells, engagement of TLR4 (triggered by LPS stimulation) leads to proliferation and eventual production of plasma cells. However, the transcriptional signatures and molecular mechanisms that distinguish these responses are relatively unknown. Here, we employed ex vivo activation of resting murine splenic B cells to examine these molecular signatures and define the transcriptional and regulatory landscape during early activation by high-resolution RNA- and ChIP-seq. We observe that at 2 h post stimulation, most genes (~90 %) induced by the two pathways are shared, despite being triggered through distinct receptors. This observation is consistent with previous studies [44, 45]. However, the rest of the transcriptome (10 %) exhibit signal-selective transcriptional programs.

While genes induced by both responses (shared) are greatly dominated by Myc targets and Myc-associated processes, groups of genes preferentially induced by LPS, as expected, show a strong correlation with NF-κB (Fig. 1). Surprisingly, BCR-induced genes showed little relationship to processes associated with the shared and LPS specific induced genes; they only overlapped with the shared-induced genes at the level of Myc and Arnt targets. Myc expression in normal lymphoid tissue is present in both activated and resting B cells in all phases of the cell cycle [46]. We were surprised to find that the Myc co-factor Max transcription levels remained constant during activation despite a 15- to 30-fold increase in Myc, suggesting other co-factors were required to drive such a strong Myc-dominated signature. However, another Myc regulator, Mnt [47], was increased by BCR activation to a small degree. These data further underscore Myc’s dominant but enigmatic role in B cell activation [48–50].

Given the distinct gene sets noted in each of the responses, we examined mechanisms that could drive these observed differences. While preexisting, paused, RNA Pol II is associated with a large number of genes in diverse cell types, new RNA Pol II recruitment indicates transcriptional activity [15]. Although preexisting RNA Pol II was found globally in resting B cells, RNA Pol II occupancy at the TSS was broadly increased during the BCR response and increased further at BCR-responsive gene promoters. This scenario suggests two general BCR-dependent mechanisms are in play [1], a global recruitment of Pol II to promoters and [2] a signal-specific guidance to intensify this general recruitment of Pol II to BCR-responsive promoters, particularly around the TSS. Conversely, LPS activation appeared to involve maintenance of steady-state RNA Pol II occupancy at the promoter relative to the resting cells. An increase in downstream RNA Pol II, possibly reflecting elongating Pol II, was noticeable, although this promoter associated RNA Pol II peak can be due to other mechanisms [51]. To better understand this phenomenon, we took a closer look at downstream regions and calculated traveling ratios of polymerase occupancy in promoter versus downstream/coding sequences (Fig. 2c). These analyses revealed that while the BCR signal resulted in enhanced RNA Pol II at promoters, the transition to downstream region (elongation) was less/slower. In contrast, although LPS stimulation did not result in additional RNA Pol II recruitment, transition to downstream regions was greater/faster than that observed with BCR. It remains possible that although the total recruitment of RNA Pol II under two stimulations is very similar, the difference in promoter versus downstream region associated RNA Pol II reflects the rate at which the enzyme transits from initiation to elongation mode. Our preliminary analysis indicates that there are differences in the complexity of gene structure (e.g., number of exons) between the BCR- and LPS-responsive genes. Whether the difference in RNA Pol II movement between the two stimuli reflects these differences, a difference in signal strength and/or a fundamental difference in signaling pathways remain to be determined.

**Fig. 7:**
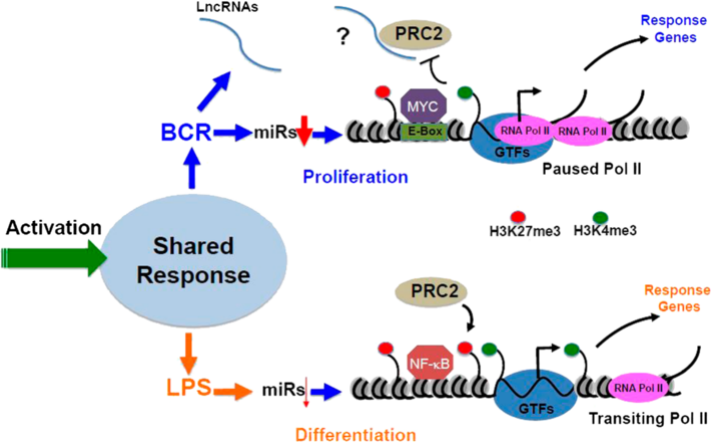
Model: Ligand-dependent regulation of B cell early activation. BCR stimulation, which eventually results in proliferation, decreases several key miRNAs compared to LPS, which leads to differentiation. BCR signaling results in greater recruitment of RNA Pol II that appeared to be paused and a decrease in H3K27me3; the latter likely achieved by displacement of PRC2. BCR activation also induces certain long non-coding RNAs (lncRNA); it is possible that binding of PRC2 to lncRNAs results in its removal from the TSS region to decrease H3K27me3. BCR-induced genes have a strong preference for promoter proximal E-boxes/MYC-binding sites. LPS-induced genes show a more rapid RNA Pol II transition from initiation to elongation mode, higher H3K27me3 around the TSS, and a preference for NF-κB motifs

Epigenetic regulation of gene expression plays crucial roles in lymphoid differentiation and homeostasis [23, 33, 35, 52, 53]. Although broad chromatin modification responses were seen with both BCR (demethylation of H3K27me3) and LPS (a greater increase in H3K4me3 methylation than with BCR) activation states, both of these effects were increased at response specific promoters, suggesting signal-dependent enhancement. While increased H3K4me3 during both responses was expected, a specific decrease in H3K27me3 around the TSS of BCR-induced genes is intriguing. Because EZH2/PRC2 is involved in germinal center B cell activation, where BCR signaling would be most likely to dominate [54], we speculate that PRC2 plays a role in distinguishing BCR versus LPS responses (Fig. 7). lncRNAs may guide PRC2 activity (reviewed in [55]), indicating a point of convergence between the increased lncRNA transcription and decreased H3K27me3 that we observed during BCR activation (Additional file 1: Figure S3). Conversely, the H3K27me3-specific demethylase, JMJD3/KDM6, is involved in LPS stimulation in macrophages and is recruited to the TSS [56–58]. It is thus possible that a balance of EZH2 and KDM6 counter regulation separates these two transcriptional landscapes.

Given that helix-loop-helix (HLH) transcription factors play an important role in B cell development and differentiation [59, 60], it is intriguing that HLH TF motifs are strongly represented in BCR-responsive promoters. While much work has been done on the role of E-box binding proteins in early B cell development (reviewed in [61]), the role of this class of proteins in mature B cell early activation is limited [62]. The idea that enhancer-promoter actions mediated by HLH TFs (e.g. Myc) via E-boxes might partially account for the increased RNA Pol II occupancy at promoters induced by BCR is appealing. A recent report that targeting BET proteins in high-risk acute lymphoblastic leukemia inhibits *Myc* and *Il7r* expression, both of which exhibit increased transcription in response to BCR in our experiment, also suggests Myc plays an important role during early phases of B cell activation [63]. The fact that E-box containing promoter sequences are underrepresented in an EZH2 recruitment assay [32] further suggests that the decrease in H3K27me3 and preponderance of E-box sequences in BCR-induced genes observed in our study are related.

As deregulation of B cell activation is related to malignancies such as B cell lymphomas, our studies may also provide insights into lymphogenesis. Myc and NF-κB are well-established master regulators of initiation of transcriptional programs, but when deregulated, they function as oncogenic drivers in B cell lymphomas. Deregulated and increased Myc and Bcl proteins, such as Bcl2 and Bcl6, are associated with particularly aggressive lymphoma types [64, 65]. Here, we found BCR stimulation decreased Bcl6 expression; in contrast, Bcl6 remained stable during LPS activation. These and other Bcl transcription patterns (Additional file 1: Figure S11) suggest that the proper regulation of Myc and Bcl proteins is required for early activation. Further investigation into this oncogenic driver network might yield interesting relationships.

Targeting of miRNAs is complex with an average miRNA having approximately 100 target sites in addition to non-canonical miRNA binding [66, 67]. Here, we identified differential expression of many miRNAs known to regulate processes involved in B cell activation. That a greater decrease in miRNA expression occurred in the BCR response compared to LPS suggests rapid downregulation of miRNAs is necessary to orchestrate gene expression driving the adaptive immune response. Given the wide activity spectrum predicted for many miRNAs, it is possible they could coordinate some of the separate regulatory mechanisms we observed. For example, a recent report proposes a regulatory loop linking overexpression of Myc, EZH2, and miR26a repression to lymphoma growth [68]. Our BCR activation data showing overexpression of Myc, lowered H3K27me3, and decreased miR26a highlight the multi-factorial nature and cross-dependency of regulatory systems likely to drive complicated responses such as signal-specific B cell activation. Although miRNAs have generally been associated with oncogenic pathways, targeted deletion of miR-17 cluster shows defects in B cell differentiation [39, 69]. Because LPS but not BCR signaling in splenic B cells results in differentiation, it is tempting to speculate that signal-specific regulation in the miR-17 cluster is a way of distinguishing between the two signals. The miR-15 cluster belongs to a very selective group of miRNAs enriched in the nucleus and thus capable of further directly regulating LPS specific transcription [70]. Despite the fact that miRNAs are critical regulators of diverse biological processes, differential regulation of miRNAs to the extent observed in our analysis is very surprising. However, it is currently unknown if these miRNAs are regulated by rapid turnover of miRNAs, regulated at the level of transcription, or both.

BCR responses are slower and presumably more precise [44, 45, 71], therefore, it is tempting to speculate that a tighter regulatory environment is required to orchestrate these lengthy responses (Fig. 7). An increase in global Pol II recruitment that appears to be regulated at the level of pausing and a greater release of miRNA repression could reflect this strict regulation. Conversely, TLR4-mediated signaling is reflective of innate responses, which are generally rapid and transient, and therefore could be manifested by an enhanced transition of RNA Pol II from initiation to elongation together with a global increase in activation marks at TSSs. Additionally, one would expect the derepression (via decreasing H3K27me3) observed during the BCR response to be slower than the sharper increase in preexisting H3K4me3-activating marks observed during LPS/TLR4 signaling. How these different observations are related to each other will be the next challenging phase to understand the regulation of B cell activation. Nevertheless, our observations begin to elucidate the signal-specific signatures involved in early activation of B cells and further suggest key molecular mechanisms (Fig. 7) that govern this important process.

## Conclusions

We conclude that the B cell commitment to different activation states is dependent upon rapid regulatory mechanisms and occurs much earlier than previously thought. Different RNA Pol II recruitment and transition from initiation to elongation, distinct activating (H3K4me3) and repressing (H3K27me3) histone signatures, mutually exclusive transcription factor binding in promoters and highly selective miRNA profiles distinguish these responses.

## Methods

### Cells and induction

Naïve resting B cells from splenocytes of 8-week-old male C57BL6 mice were isolated with anti-CD43 beads (Miltenyi), confirmed as 95 % CD19^+^ by flow cytometry (FACS Calibur), and resuspended in cold media with either 10 ug/ml anti-mouse IgM goat IgG Fab fragments (Jackson Immunology) or 25 ug/ml Salmonella typhimurium typhus LPS (Sigma) were added. The cells were rested on ice for 30 min following a previously published method [72] and incubated at 37 °C/5 %CO_2_ for the experimental times. Animal care and use in this study are covered under the “Assurance of Compliance with PHS (USA) Policy on Humane Care and Use of Laboratory animals by Awardee Institutions” and approved by the Institutional Animal Care and Use Committee of Tufts University (Animal Welfare Assurance Number A-3775-01).

### Deep sequencing

Sample preparation was performed using common techniques. In general, single end, 100 bp (initial RNA), and 50 bp (secondary RNA, ChIP and miRNA) reads were mapped against the mm9/ENSEMBL build 67 genome reference using Tophat v2.0.0 [73] and for RNA, bowtie 1.0.0 for ChIP [74]. RNA Pol II ChIP-seq employed antibody against total RNA Pol II (Santa Cruz N-20, sc-816x), H3K4me3 with Abcam antibody ab8580, and H3K27me3 with Abcam antibody ab6002. Mapped read numbers per million and BCR or LPS time points are 120 min unless indicated. RNA [1], rest 75.3, BCR30 20.9, BCR120 73.6, LPS30 44.7, LPS120 40.5; RNA [2], rest 77, BCR 61, LPS 68.9; RNA Pol II, rest 18.1, BCR 14.2, LPS 21.3; H3K4me3, rest 13.1, BCR 16.4, LPS 19.1; K3K27me3, rest 19.0, BCR 20.0, LPS 18.1.

### Differential expression analysis

Differential expression (DE) was identified by a minimal twofold difference in log ratios of normalized reads generated with Cufflinks v1.3.1. Preferentially induced or reduced genes sets included genes that were either changed by either a single response or when affected by both responses changed only two- to fourfold by one response and were changed by the preferred response at a ratio of at least twofold more than the non-referred response. A spreadsheet of the differential expression list can be found in Additional file 3. Genes were annotated to biological processes with the online Toppfun program. Gene network analysis was carried out using ToppCluster [75] and visualized by Cytoscape [76].

### miRNA-seq analysis

After Trizol isolation of RNA, TruSeq Small RNA Sample Preparation Kits were used to produce material for generating 50 bp single end reads which were then analyzed with miRDeep2 [77] using the miRBase reference v14 with standard settings. Mapped miRNAs were confirmed by visual inspection of miRNA structure and UCSC Genome Browser tracks [78] and inclusion in the Ensembl data base [79]. Differential expression from the resting state was identified by a minimal twofold difference in miRdeep2 normalized reads. Total miRNA data set reads per million are the following: rest 20.2, BCR 120 28.6, and LPS 120 14.8. Total miRdeep2 miRNA reads (per thousand) are the following: rest 55.8, BCR 16.2, and LPS 27.6.

### ChIP-seq analysis

For histograms of TSS coverage, custom R scripts were used to produce bedgraphs from mapped bam files, which were converted to BigWig files with bedgraphToBigwig for UCSC Genome Browser presentation [80, 81]. Reads per million-normalized coverage was computed for the gene sets and regions indicated, and summary statistics were calculated at each base pair, for histograms or by summing total coverage across regions, as shown for boxplots. Traveling ratios (TR) were calculated from the mean of summed transcript RPM means for each transcription group in an area representing the promoter (p) (−0.3/0.3 kbp) and downstream body (b) (0.3/2.25 kbp) of the transcript, TR = (b/p). TRs were then normalized to the resting state (TRactivation/TRrest).

### CpG and TF motif analysis

Predicted CpG island locations were from preloaded USCS Genome Browser tracks and produced by common methods [82]. Proximity of TSSs to CpG islands was analyzed with Bedtools’ IntersectBed [80]. Enriched TF binding motifs in the promoters, defined as −1000 to +1000 regions relative to the TSS based on RNA Pol II occupancy (Fig. 2), employed the motif enrichment algorithm implemented in the HOMER tool [83] supplemented with the mouse TF binding motifs contained in the CisBP database (build 0.90) [84], resulting in a total of 3812 mouse motifs. Enrichment calculations used promoter sequences of genes whose expression did not change as our background set.

### Quantitative PCR-RNA validation

Real-time PCR was performed with specific primers (Additional file 1: Figure S10) using previous methods [85]. Target sequences are reported relative to Beta Actin and normalized to resting cells.

### GEO datasets

The sequences have been deposited to the GEO database (NCBI/NLM/NIH)—accession number (GSE61608) (http://www.ncbi.nlm.nih.gov/geo/query/acc.cgi?acc=GSE61608).

## Additional files

Additional file 1: Figures S1–S11.
**Figure S1.** 120 min is required for recognizably organized response. Figure S2. Characterization of unchanged transcription. **Figure S3.** Stimulus specific of high polymerase occupancy. **Figure S4.** RNA Pol II data for each cellular state. **Figure S5.** Global RNA Pol II recruitment and histone changes. **Figure S6.** Stimulus-specific histone methylation at high Pol occupancy TSS(s). **Figure S7.** Closer examination of individual BCR induced gene tracks to better show examples of H3K27me3 reduction upon BCR activation. **Figure S8.** Lack of CpG-dependent effect on Pol II occupancy. **Figure S9.** Expression of activation-responsive miRNAs. **Figure S10.** Observation of lncRNA Malat1 behavior with validation and correlating activation regulation mechanisms. **Figure S11.** Response-dependent difference in Bcl network. Additional file 2:
**Genes involved in NF-κB signaling.** A list of genes induced by either BCR or LPS, or both (shared), found in the KEGG Pathway NF-κB signaling pathway. Additional file 3:
**Differentially expressed genes and miRNAs.** Table S1: a list of genes with statistically significant differential expression calculated as described in Methods. Table S2: a list of miRNAs with statistically significant differential expression calculated as described in Methods.
