# Supplementary material for: Divergence of transcriptional landscape occurs early in B cell activation
Source: Epigenetics Chromatin. 2015 May 14;8:20. doi: 10.1186/s13072-015-0012-x (PMC4434543; doi:10.1186/s13072-015-0012-x)
Supplement: Additional file 1: Figures S1–S11. — Figure S1. 120 min is required for recognizably organized response. Figure S2. Characterization of unchanged transcription. Figure S3. Stimulus specific of high polymerase occupancy. Figure S4. RNA Pol II data for each cellular state. Figure S5. Global RNA Pol II recruitment and histone changes. Figure S6. Stimulus-specific histone methylation at high Pol occupancy TSS(s). Figure S7. Closer examination of individual BCR induced gene tracks to better show examples of H3K27me3 reduction upon BCR activation. Figure S8. Lack of CpG-dependent effect on Pol II occupancy. Figure S9. Expression of activation-responsive miRNAs. Figure S10. Observation of lncRNA Malat1 behavior with validation and correlating activation regulation mechanisms. Figure S11. Response-dependent difference in Bcl network. [file 13072_2015_12_MOESM1_ESM.pdf]

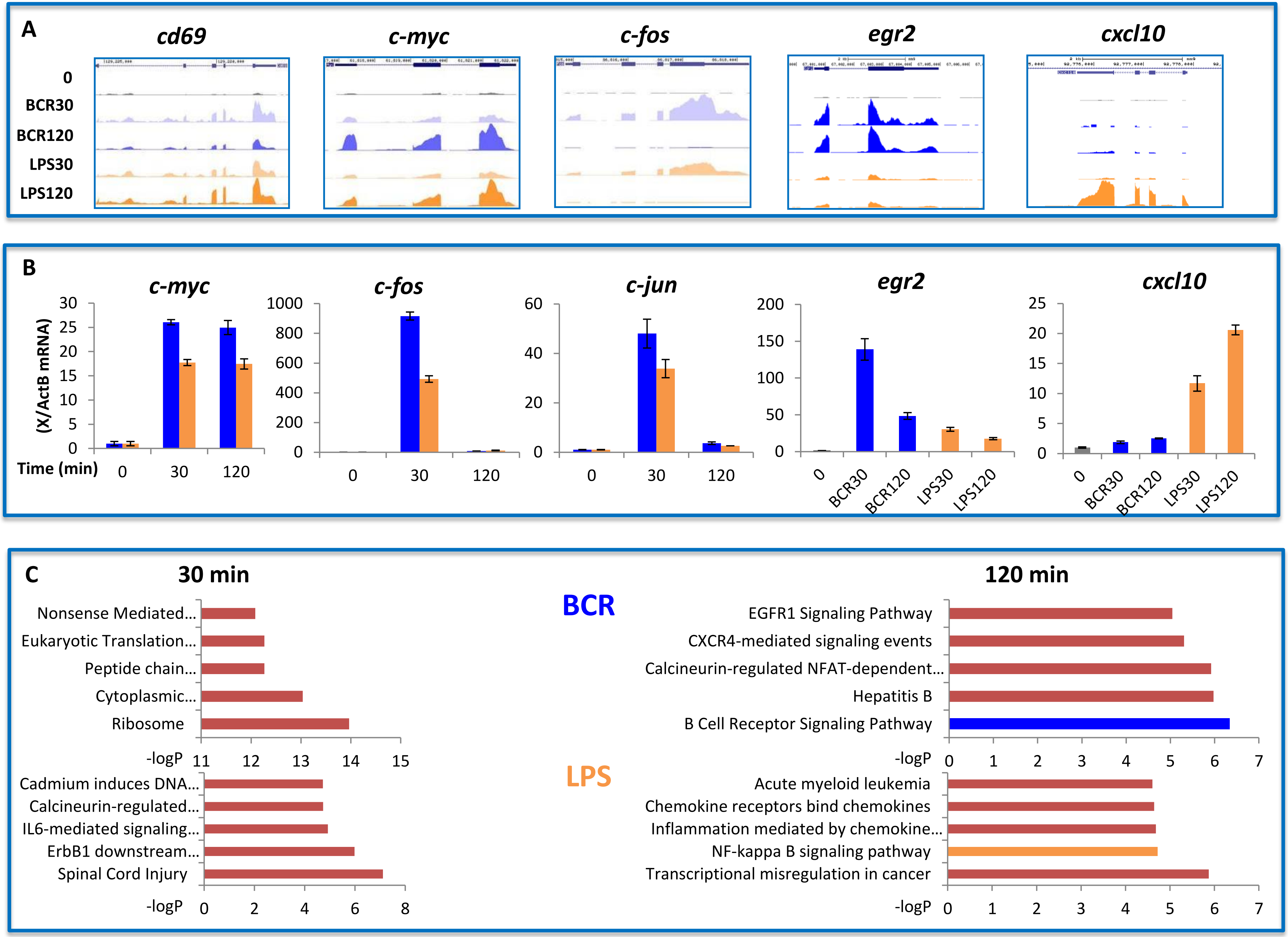

A
cd69
c-myc
c-fos
egr2
cxcl10
0
BCR30
BCR120
LPS30
LPS120
B
c-myc
c-fos
c-jun
egr2
cxcl10
(X/ActB mRNA)
Time (min)
0
30
120
0
BCR30
BCR120
LPS30
LPS120
C
30 min
120 min
BCR
LPS
Nonsense Mediated...
Eukaryotic Translation...
Peptide chain...
Cytoplasmic...
Ribosome
EGFR1 Signaling Pathway
CXCR4-mediated signaling events
Calcineurin-regulated NFAT-dependent...
Hepatitis B
B Cell Receptor Signaling Pathway
Cadmium induces DNA...
Calcineurin-regulated...
IL6-mediated signaling...
ErbB1 downstream...
Spinal Cord Injury
Acute myeloid leukemia
Chemokine receptors bind chemokines
Inflammation mediated by chemokine...
NF-kappa B signaling pathway
Transcriptional misregulation in cancer
-logP

Fig S1

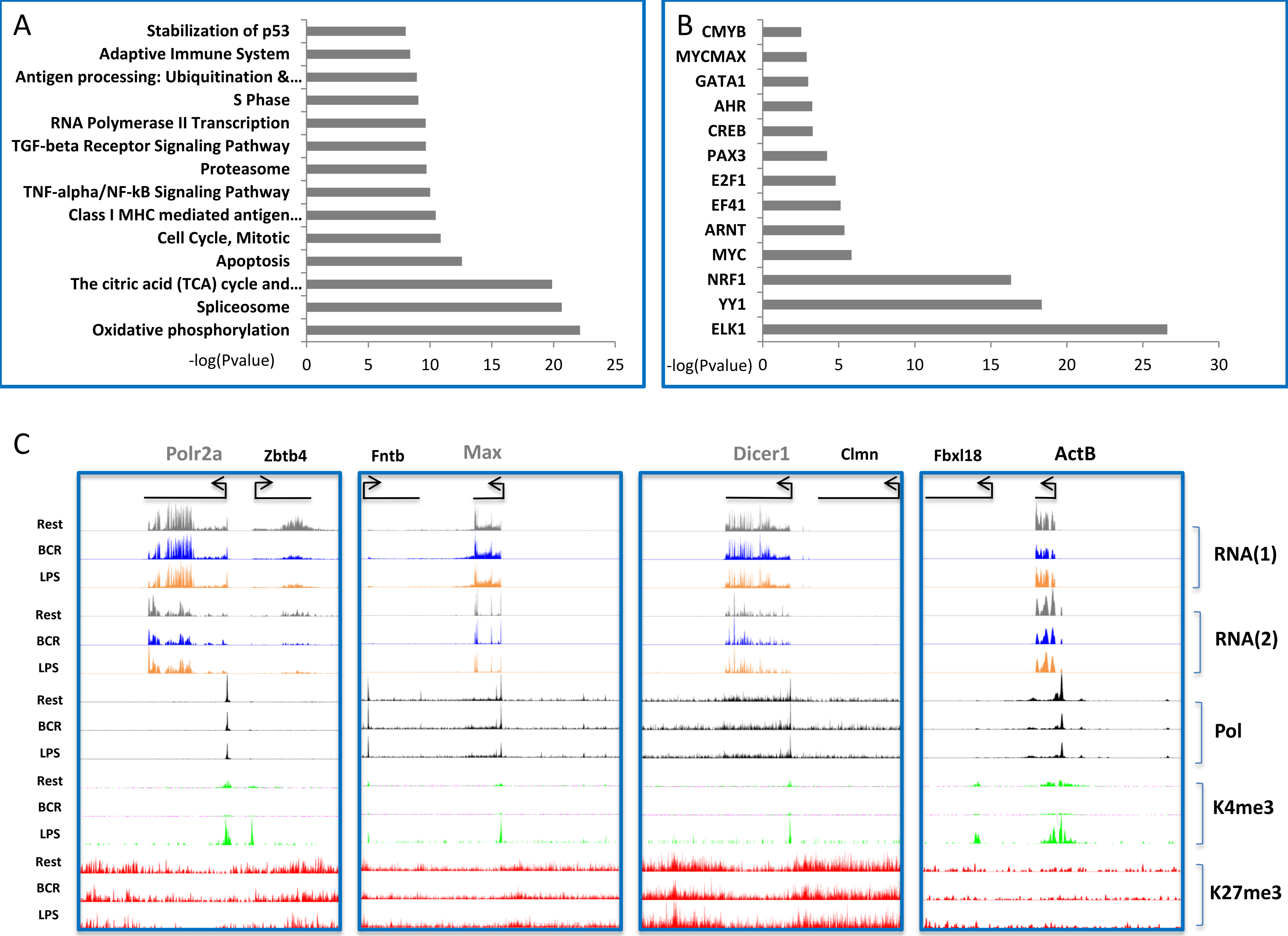
A
Stabilization of p53
Adaptive Immune System
Antigen processing: Ubiquitination &...
S Phase
RNA Polymerase II Transcription
TGF-beta Receptor Signaling Pathway
Proteasome
TNF-alpha/NF-kB Signaling Pathway
Class I MHC mediated antigen...
Cell Cycle, Mitotic
Apoptosis
The citric acid (TCA) cycle and...
Spliceosome
Oxidative phosphorylation
-log(Pvalue)
0 5 10 15 20 25
B
CMYB
MYCMAX
GATA1
AHR
CREB
PAX3
E2F1
EF41
ARNT
MYC
NRF1
YY1
ELK1
-log(Pvalue)
0 5 10 15 20 25 30
C
Polr2a
Zbtb4
Fntb
Max
Dicer1
Clmn
Fbxl18
ActB
Rest
BCR
LPS
RNA(1)
RNA(2)
Pol
K4me3
K27me3

Fig S2

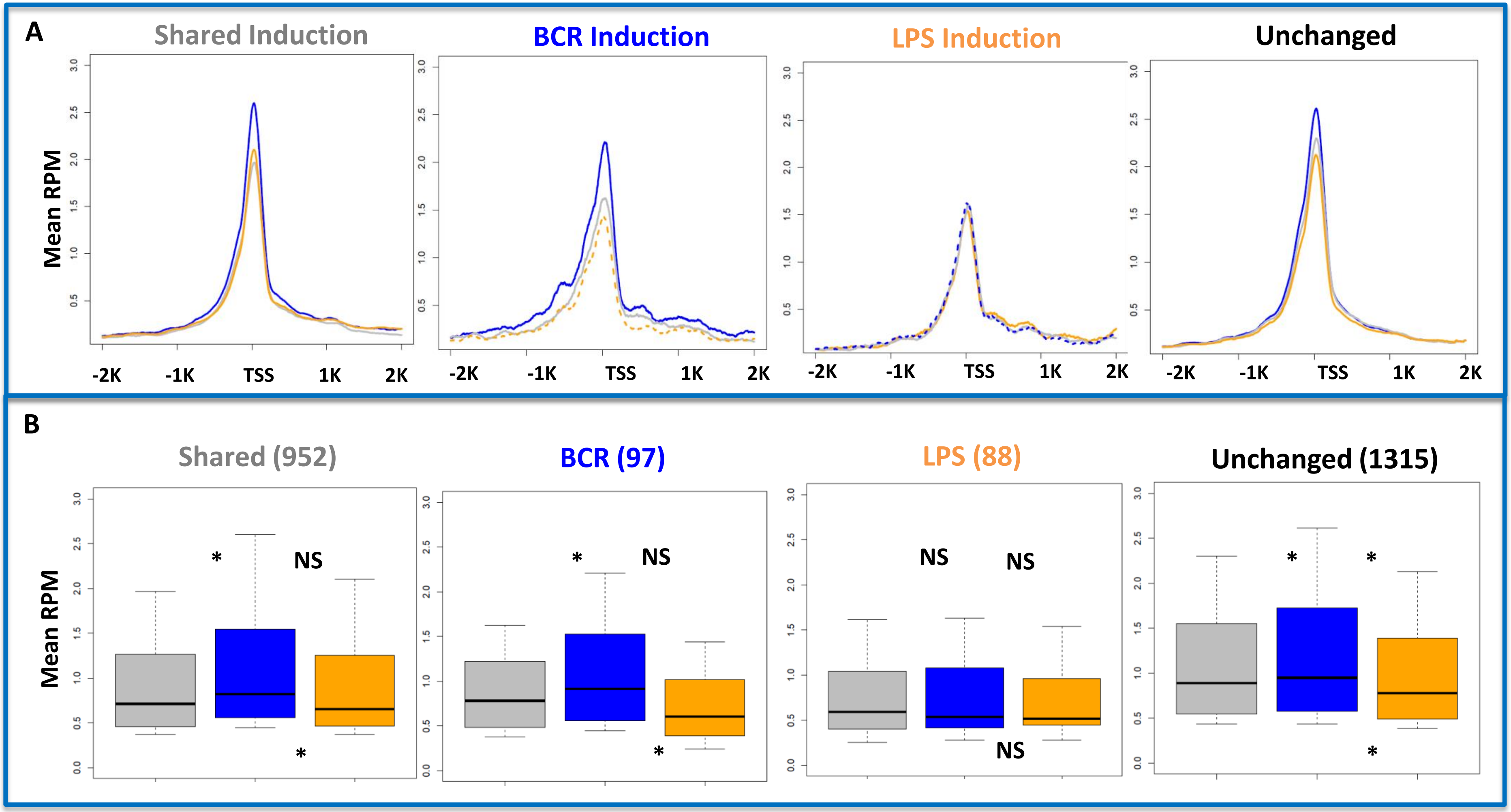
A
Shared Induction
BCR Induction
LPS Induction
Unchanged
Mean RPM
-2K
-1K
TSS
1K
2K
B
Shared (952)
BCR (97)
LPS (88)
Unchanged (1315)
Mean RPM
*
NS
NS

Fig S3

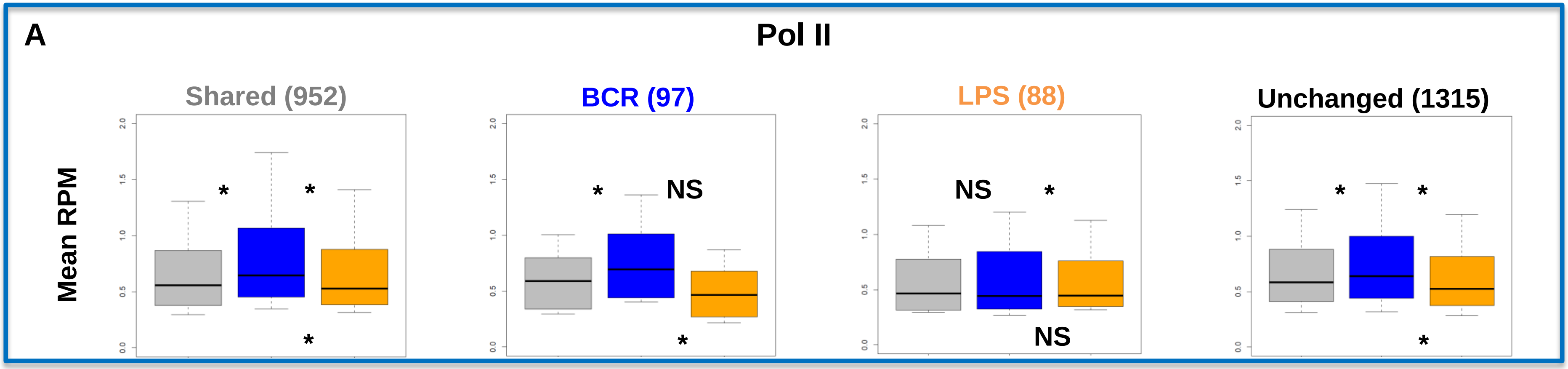
A
Pol II
Shared (952)
BCR (97)
LPS (88)
Unchanged (1315)
Mean RPM
*
NS

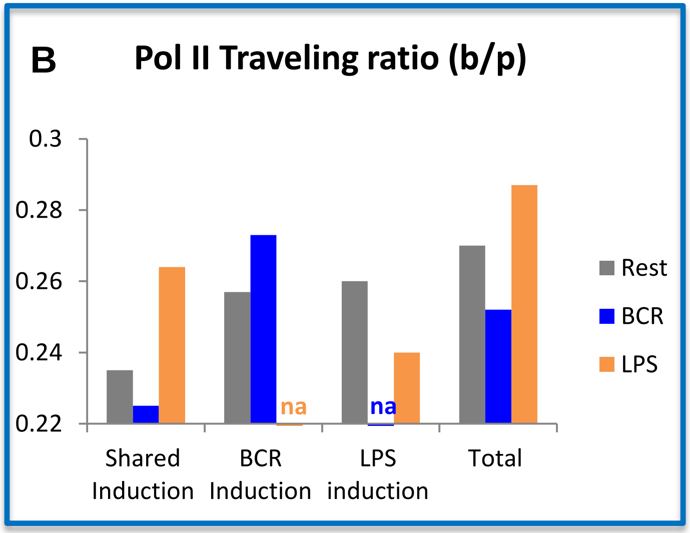
B
Pol II Traveling ratio (b/p)
0.3
0.28
0.26
0.24
0.22
na
Shared Induction
BCR Induction
LPS induction
Total
Rest
BCR
LPS

Fig S4

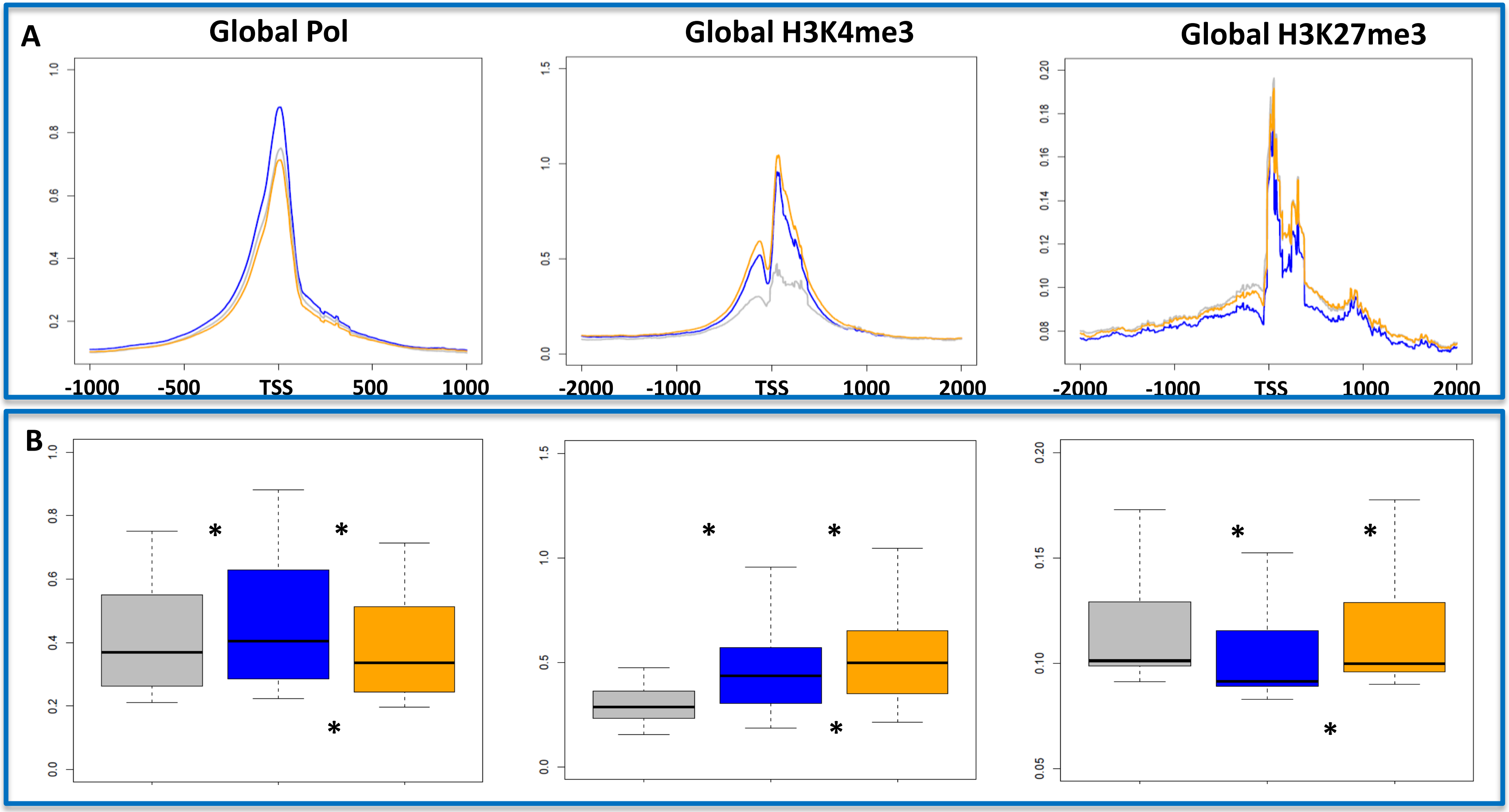
A
Global Pol
Global H3K4me3
Global H3K27me3
-1000
-500
TSS
500
1000
-2000
-1000
TSS
1000
2000
-2000
-1000
TSS
1000
2000
B
*
*
*
*
*
*
*
*
*

Fig S5

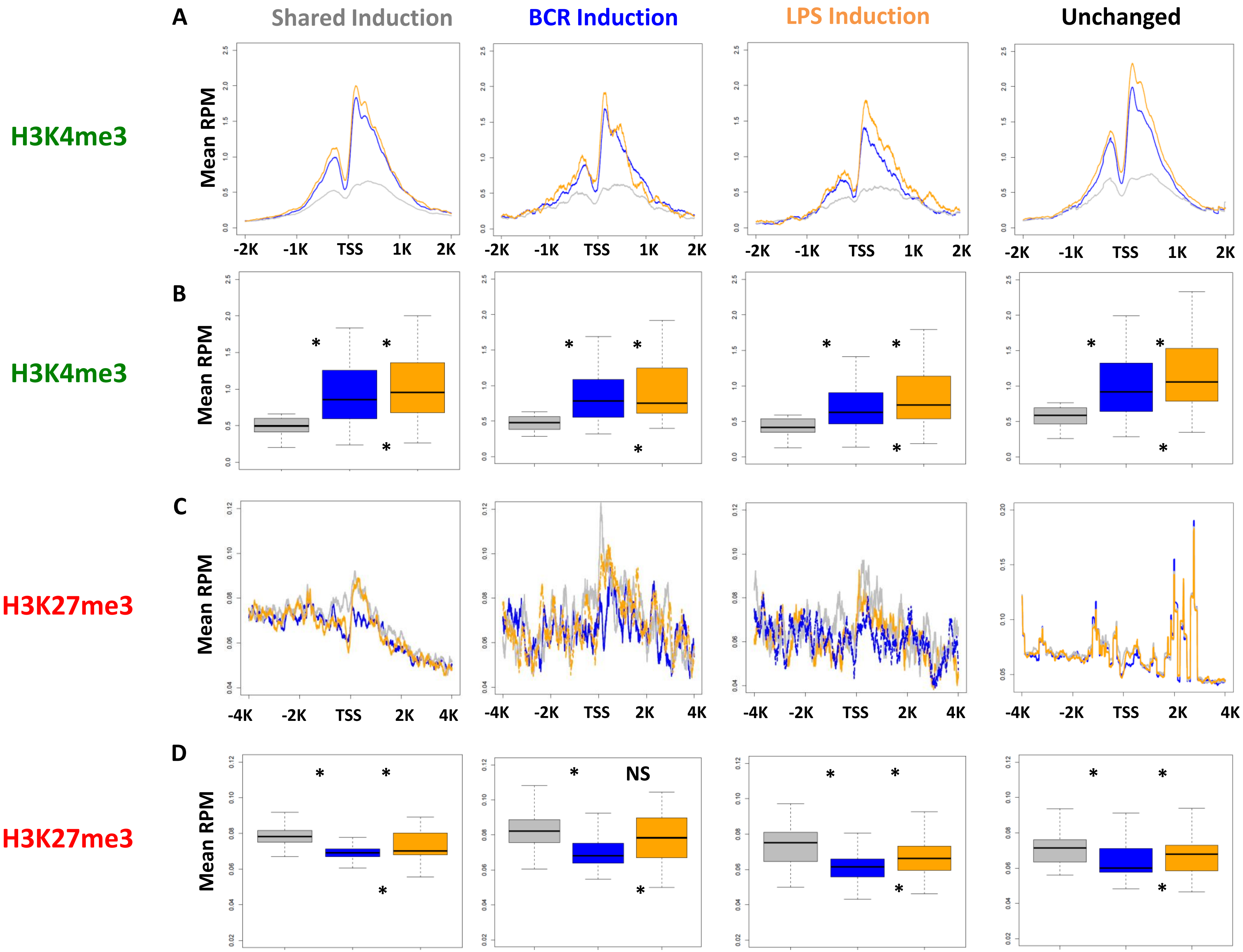

A
Shared Induction
BCR Induction
LPS Induction
Unchanged
H3K4me3
Mean RPM
-2K
-1K
TSS
1K
2K
B
H3K4me3
Mean RPM
*
NS
C
H3K27me3
Mean RPM
-4K
-2K
TSS
2K
4K
D
H3K27me3
Mean RPM

Fig S6

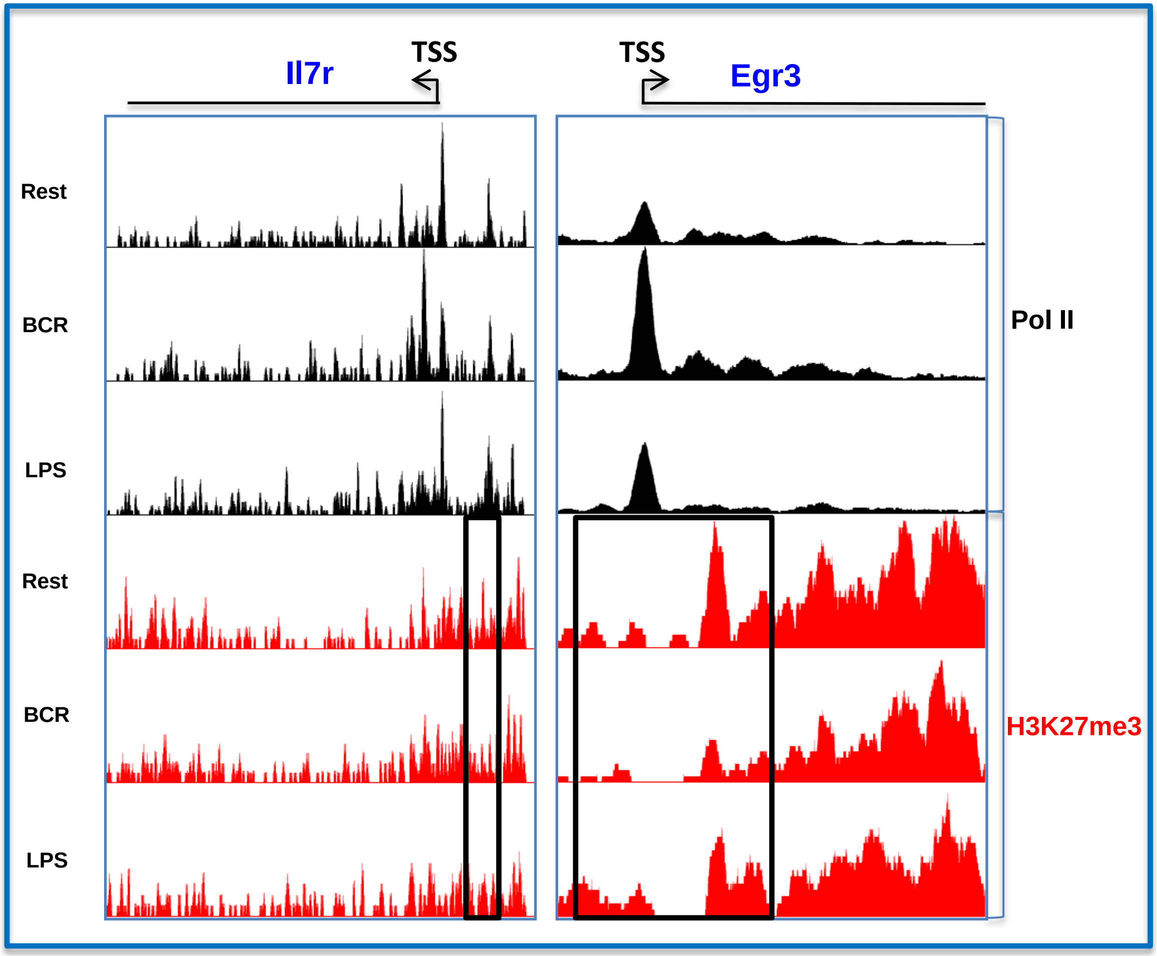

Fig S7

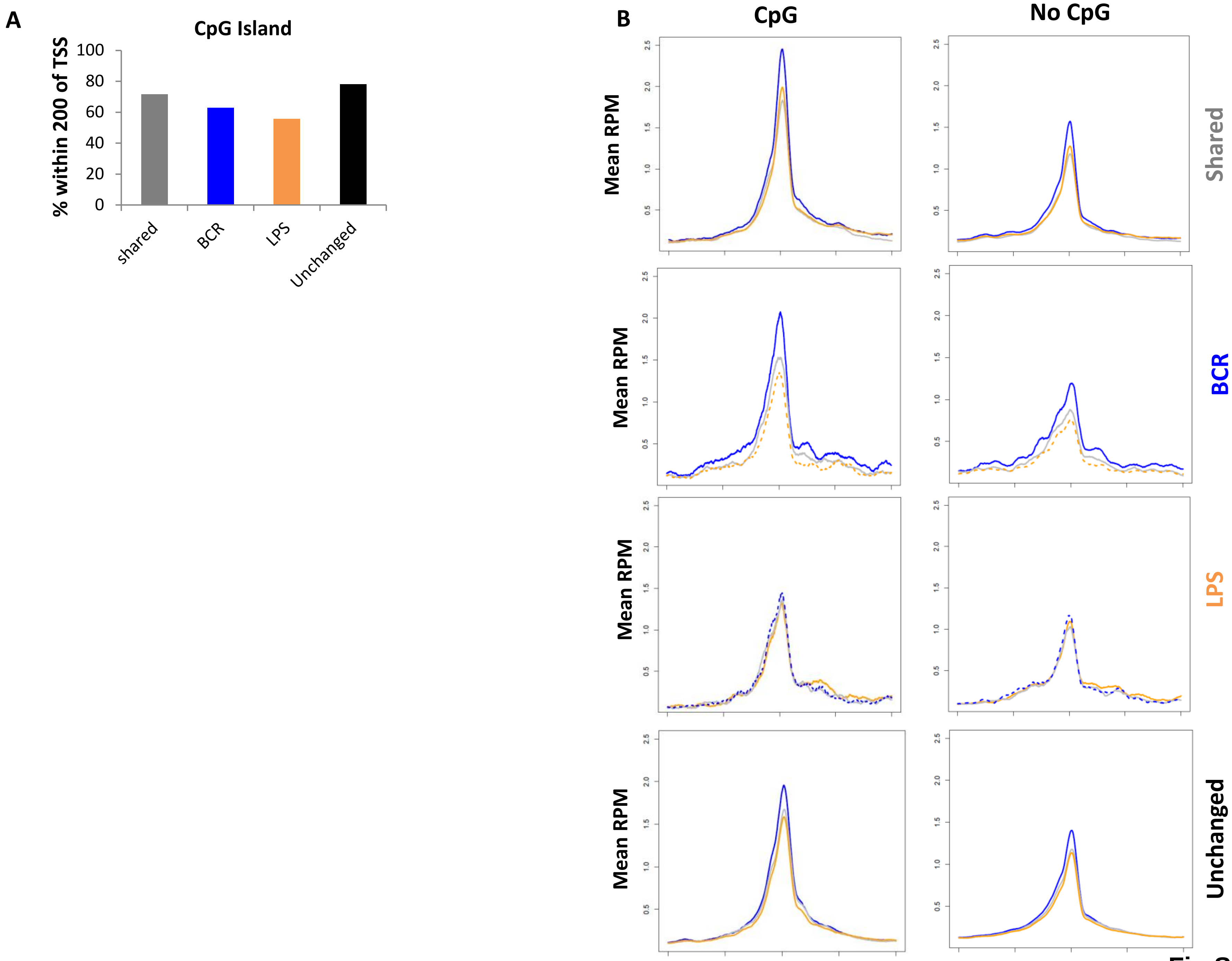

A
CpG Island
% within 200 of TSS
100
80
60
40
20
0
shared
BCR
LPS
Unchanged
B
CpG
No CpG
Mean RPM
Shared
BCR
LPS
Unchanged

Fig S8

miRNA supplemental

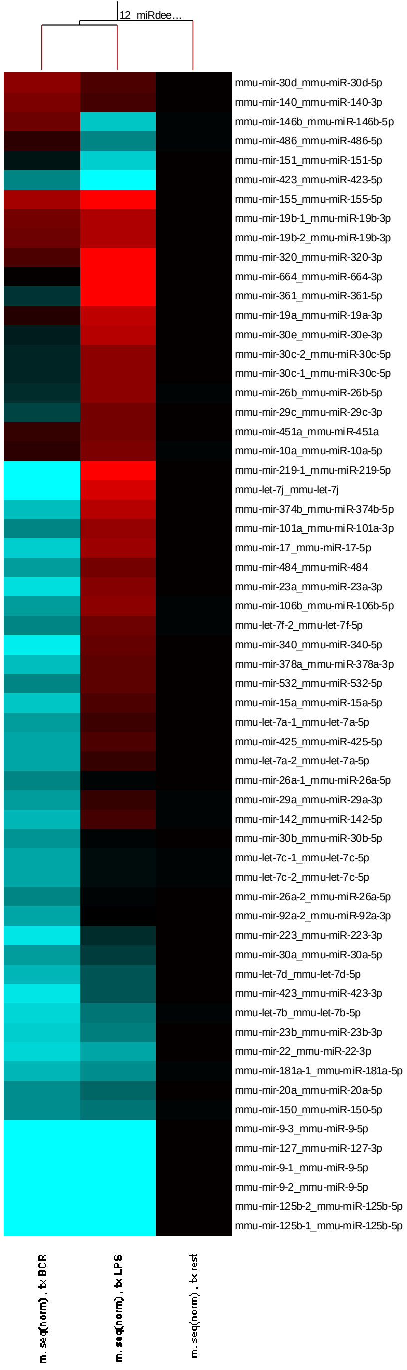

Fig S9

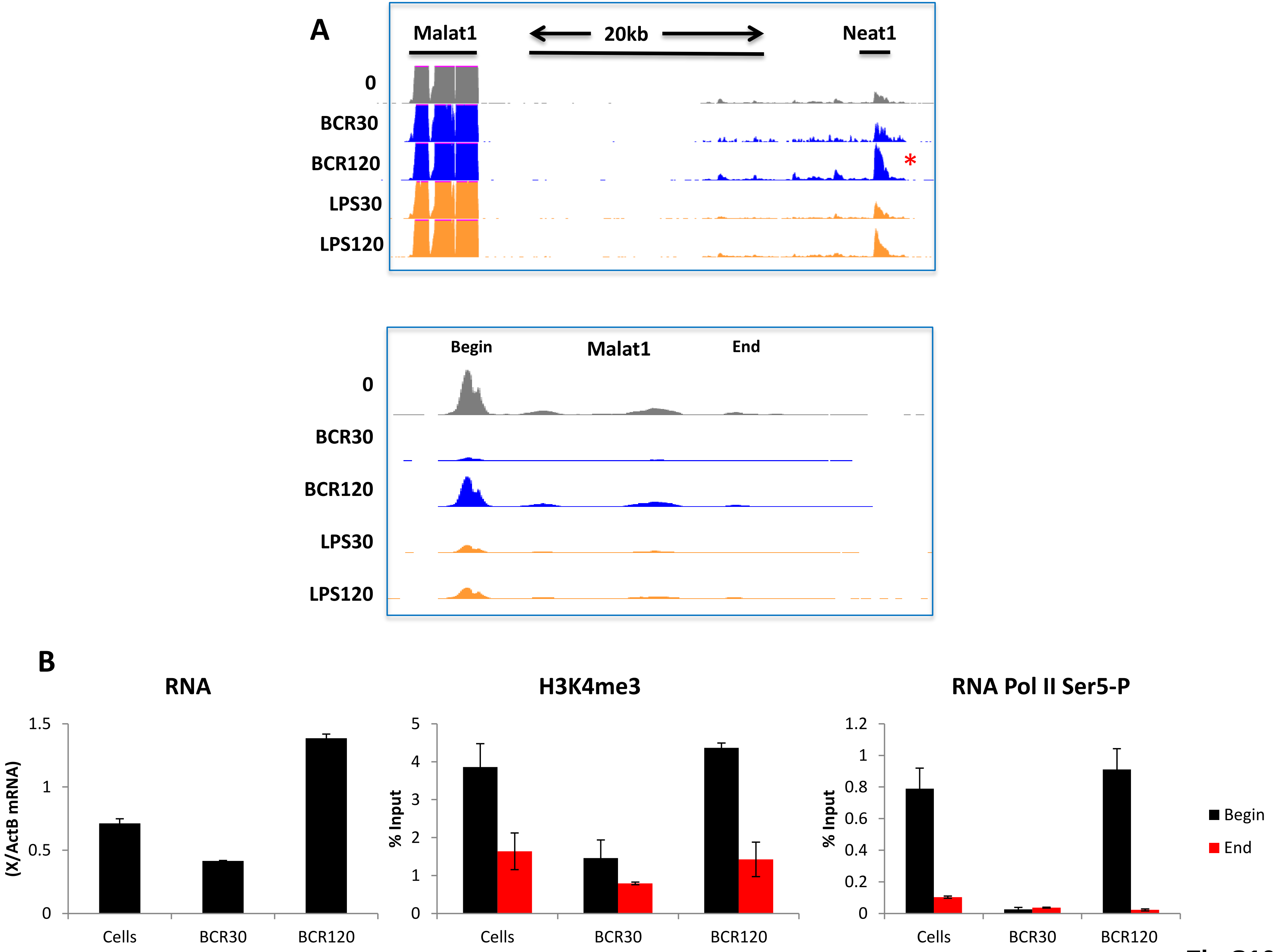
A
Malat1
20kb
Neat1
0
BCR30
BCR120
*
LPS30
LPS120
Begin
Malat1
End
0
BCR30
BCR120
LPS30
LPS120
B
RNA
(X/ActB mRNA)
1.5
1
0.5
0
Cells
BCR30
BCR120
H3K4me3
% Input
5
4
3
2
1
0
Cells
BCR30
BCR120
RNA Pol II Ser5-P
% Input
1.2
1
0.8
0.6
0.4
0.2
0
Cells
BCR30
BCR120
Begin
End

Fig S10

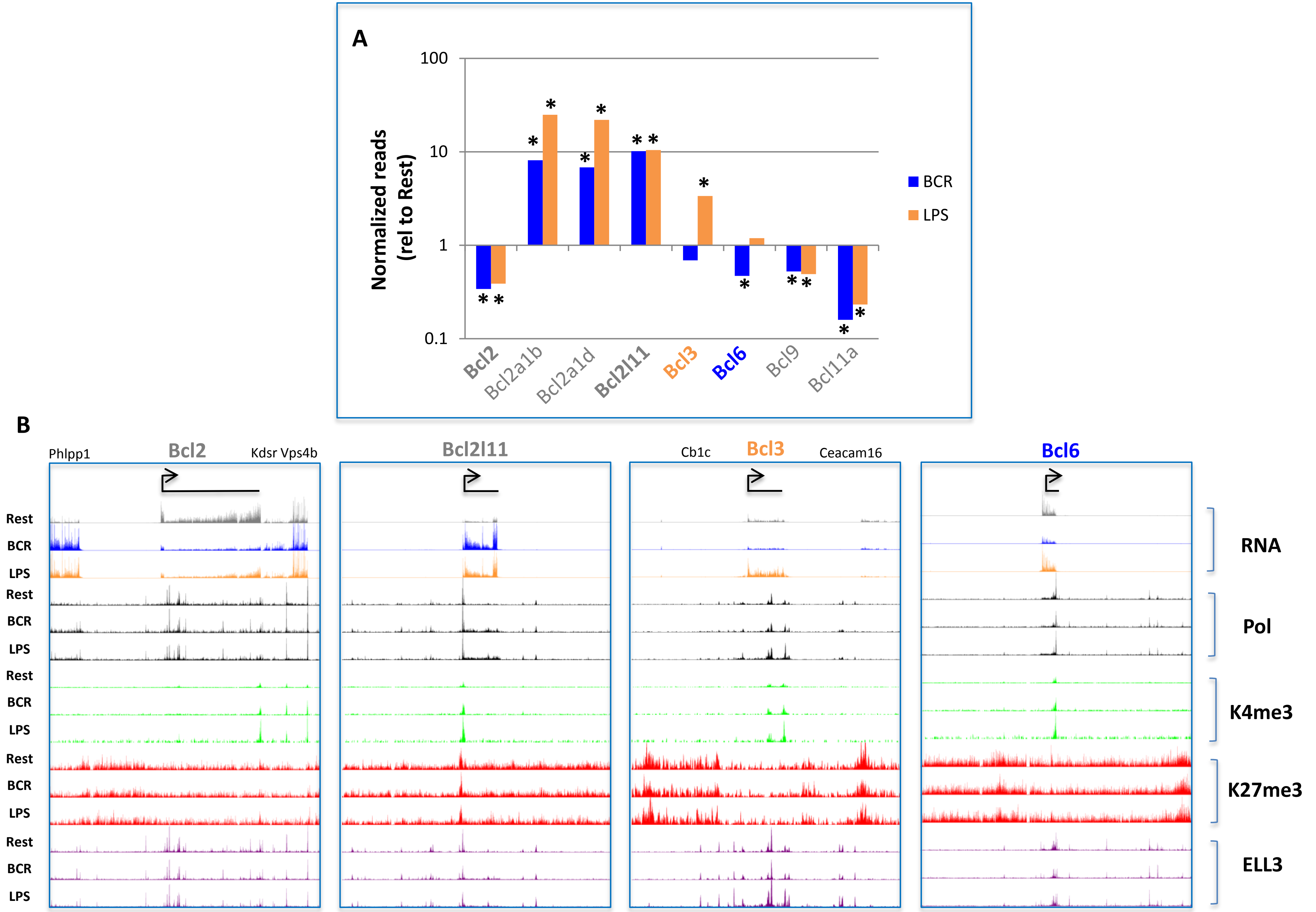
A
100
10
1
0.1
Normalized reads
(rel to Rest)
BCR
LPS
Bcl2
Bcl2a1b
Bcl2a1d
Bcl2l11
Bcl3
Bcl6
Bcl9
Bcl11a
B
Phlpp1
Bcl2
Kdsr Vps4b
Bcl2l11
Cb1c
Bcl3
Ceacam16
Bcl6
Rest
BCR
LPS
RNA
Pol
K4me3
K27me3
ELL3

Fig S11

Supplementary figure legends.

Supplemental Fig S1. 120 minutes is required for recognizably organized response. UCSC Genome Browser tracks showing activation of genes associated with activation at 30 and 120 minutes **(A)** and **(B)** RT-PCR confirmation of engaged primary response gene transcription at 30 and 120 minutes. **(C)**. Genes with significantly increased expression as measured by Cuffdiff analysis were analyzed for pathway associated transcription with ToppFun. Number of genes differentially expressed, as determined by Cuffdiff with standard settings; BCR30=65, LPS30=41, BCR120=288, LPS120=90. The top 5 pathways are shown with the corresponding log of P value.

Supplemental Fig S2. Characterization of unchanged transcription. 1315 genes with significant levels of transcription (FPKM>10) at resting which did not change significantly during BCR or LPS induction (0.75<x>1.25). These genes were used as the Unchanged group through the study as a baseline. **(A)**. Analysis of gene list with Toppfunn showing pathways (KEGG or Reactome) with significant overlap with the genes. Selected pathways are shown with corresponding –log p value. **(B)**. Transcription factor binding sites associated with unchanged genes. **(C)**. UCSC Genome Browser tracks of selected unchanged genes. ActB, not included in the unchanged list, is also shown as it was used as a control in RT-PCR validation.

Supplemental Fig S3. Stimulus specific of high Polymerase occupancy. **(A).** For each of the responsive and unchanged genes, the TSS with the highest Pol occupancy in the resting state was identified. Normalized reads of Pol occupancy were mapped 2000 bp on either side of this high occupancy transcription start site (TSS) of genes with increased transcription shared between the two activation states (far right), exclusive to BCR (middle right), exclusive to LPS (middle left) and in genes with substantial but unchanged transcription relative to the resting state. Reads from cell in the resting state are grey, BCR activation state in blue, LPS activation state in orange. **(B)**. Boxplots contain the sums of mapped normalized mean reads per million (RPM) +/-500 TSS. Resting=grey, BCR=blue,LPS=orange. Non-significant (p= or >0.05) or significant (*) from Wilcoxon Rank Sum Testing are indicated and given here in the following order; shared(rest/bcr)<2e-16, shared(rest/lps)=0.96, shared(bcr/lps)<2e-16, bcr(rest/bcr)<2e-16, bcr(rest/lps)=2e-16, bcr(bcr/lps)<2e-16, lps(rest/lps)=0.33, lps(rest/bcr)=0.66, lps(lps/bcr)=0.88,unchanged(rest/bcr)=6e-4, unchanged(rest/lps)=2e-7, unchanged(bcr/lps)<2e-15. * or NS on top refers to difference from the resting state and when at bottom a significant difference between the BCR and LPS state.

Supplemental Fig S4. RNA Pol II data for each cellular state. (A). Boxplots contain the sums of mapped normalized mean RPM +/-500 bases relative to average TSS; resting (grey), BCR (blue), LPS (orange), number of genes indicated in parentheses. (NS) Non-significant (p >= 0.05) or significant (*, p <0.05) from Wilcoxon Rank Sum Testing. (*) or NS on top indicates a difference from the resting state, and at bottom between the BCR and LPS states. **(B).** Traveling ratios for RNA Pol II. Traveling ratios of Pol II occupancy were determined to describe average RNA Pol II movement between the promoter and interior of the gene. Ratios of mean RPMs. shown at black line in boxplot from (Fig 2B), were calculated; traveling ratio (TR)=(RPM mean)body(b)/(RPM mean)promoter (p).

Supplemental Fig S5 Global RNA Pol II recruitment and histone changes. (A). Histograms of normalized mean read coverage around global TSS for Pol II (left), H3K4me3 (center), and H3K27me3 (right). **(B).** Boxplot analysis of normalized mapped reads around total TSS for Pol II (right, 500 bp), H3K4me3 (1000 bp, center), and H3K27me3 (1000 bp, right). Resting=grey, BCR=blue,LPS=orange. Non-significant (p= or >0.05) or significant (*) from Wilcoxon Rank Sum Testing. *=2e-16. * on top refers to difference from the resting state and when at bottom a significant difference between the BCR and LPS state.

Supplement Fig S6. Stimulus specific histone methylation at high Pol occupancy TSS(s). For each of the responsive and unchanged genes, the TSS with the highest Pol occupancy in the resting state was identified. **(A)**. H3K4me3-Normalized mean reads per million (RPM) of occupancy were mapped 2000 bp on either side of transcription start sites (TSS) of genes with increased transcription shared between the two activation states (far right), exclusive to BCR (middle right), exclusive to LPS (middle left) and in genes with substantial but unchanged transcription relative to the resting state. Reads from cell in the resting state are grey, BCR activation state in blue, LPS activation state in orange. **(B).** Boxplot analysis of normalized mapped reads 1000 bps around the TSS. Resting=grey, BCR=blue,LPS=orange. Non-significant (p= or >0.05) or significant (*) from Wilcoxon Rank Sum Testing are indicated and given here in the following order; shared(rest/bcr)<2e-16, shared(rest/lps)<2e-16, shared(bcr/lps)=3e-12, bcr(rest/bcr)<2e-16, bcr(rest/lps)<2e-16, bcr(bcr/lps)=7e-6, lps(rest/bcr)<2e-16, lps(rest/lps)<2e-16, lps(lps/bcr)<2e-16,unchanged(rest/bcr)<2e-16, unchanged(rest/lps)<2e-16, unchanged(bcr/lps)<2e-16. **(C)**. H3K27me3-normaliized mean reads per million 4000bp on either side of TSS. **(D).** Boxplot of H3K27me3-normalized mean reads per million 1000 bps on either side of TSS; shared(rest/bcr)<2e-16, shared(rest/lps)=2e-16, shared(bcr/lps)<2e-16, bcr(rest/bcr)<2e-16, bcr(rest/lps)=0.85, bcr(bcr/lps)<2e-16, lps(rest/lps)<2e-16, lps(rest/bcr)<2e-16, lps(lps/bcr)<2e-16,unchanged(rest/bcr)<2e-16, unchanged(rest/lps)<2e-16, unchanged(bcr/lps)<2e-14. * or NS on top refers to difference from the resting state and when at bottom a significant difference between the BCR and LPS state.

Supplemental Fig S7. **Closer examination of individual BCR induced gene tracks to better show examples of H3K27me3 reduction upon BCR activation**. Il7r, left, shows a modest decrease at a point proximal to the TSS for both BCR and LPS while Egr3, right, shows an activation related decrease much greater in the BCR response.

Supplemental Fig S8. Lack of CpG dependent effect on Pol II occupancy. (A). % of promoters intersecting or within 200 bp of predicted CpG islands**. (B).** Histograms of normalized Pol II ChIP read coverage around +/-1000bp of the TSS of responsive genes that whose TSS is (CpG) intersecting or within 200bp of a predicted CpG island or not (No CpG). Analysis via Wilcoxon Rank Sum test showed statistically significant change between all CpG and non-CpG promoter occupancies in each response.

Supplemental Fig S9. **Expression of activation responsive miRNAs**. miRNAs as analyzed by the program miRdeep2 were considered if the raw read count at resting was 10 or above as analyzed by mideep2 with standard settings. For selected miRNAs, their miRdeep2 normalized read counts underwent K-means clustering with Euclidean distance metrics using the Cluster 3.0 program. Heat map visualization of clusters was performed with Java TreeView.

Supplemental Fig S10. Observation of lncRNA Malat1 behavior with validation and correlating activation regulation mechanisms. **(A)**. Visualization of transcription of mapped reads at Malat1 and Neat1 from UCSC Genome Browser. * denotes significant increase in transcription in duplicate 120 minute data sets in duplicate RNA sequencing analysis as in Fig 1A. Below is an expanded view of the detectable read pattern seen for Malat1. **(B)**. RT-PCR analysis of Malat1 (at beginning) during early BCR activation for RNA, reported as the ratio of ActinB mRNA, and chromatin immunoprecipitation of H3K4me3 (middle), and "paused" RNA Pol II-Ser5-P (right) at the beginning and end (locations indicated in A) of Malat1.

Supplemental Fig S11. Response dependent difference in Bcl network. **(A)** B-cell CLL/lymphoma (Bcl) protein transcription normalized RNA reads were set relative to readings found in the resting state and graphed. * denotes a minimal two fold change relative to the resting state with a Student's T test p value <0.05. **(B)** UCSC Genome Browser tracks of selected Bcl family members with response shared decrease (Bcl2), shared increase (Bcl2l11), lps increase (Bcl3), and BCR decrease (Bcl6).
